# Chemo-Immunotherapy Regimes for Recurrent or Metastatic Nasopharyngeal Carcinoma: A Network Meta-Analysis and Cost-Effectiveness Analysis

**DOI:** 10.3389/fphar.2022.858207

**Published:** 2022-05-20

**Authors:** Youwen Zhu, Kun Liu, Dong Ding, Kailing Wang, Xiaoting Liu, Xiao Tan

**Affiliations:** ^1^ Department of Oncology, Xiangya Hospital, Central South University, Changsha, China; ^2^ Department of Oncology, Enshi Central Hospital, Wuhan University, Hubei, China; ^3^ Department of Gastroenterology, Xiangya Hospital, Central South University, Changsha, China; ^4^ Health Management Center, Brain Hospital of Hunan Province, Changsha, China

**Keywords:** recurrent or metastatic nasopharyngeal carcinoma, toripalimab, camrelizumab, gemcitabine and cisplatin, cost-effectiveness

## Abstract

**Introduction:** In 2021, two phase III clinical trials confirmed that toripalimab or camrelizumab combined with gemcitabine and cisplatin (TGP or CGP) provide more benefits in the first-line treatment of R/M NPC than GP. Fortunately, TGP and CGP were recently approved as first-line treatments for cases experiencing R/M NPC by the China National Medical Products Administration in 2021. However, due to the high cost and variety of treatment options, the promotion of chemo-immunotherapeutics in the treatment of R/M NPC remains controversial. Therefore, we performed a cost-effectiveness assessment of the two newly approved treatment strategies to assess which treatments provide the greatest clinical benefits at a reasonable cost.

**Methods:** A cost-effectiveness analysis and network meta-analysis network meta-analysis was conducted based on the JUPITER-02 and CAPTAIN-first Phase 3 randomized clinical trials. A Markov model was expanded for the evaluation of the effectiveness and cost of TGP, CGP, and GP chemotherapy with a 10-years horizon and measured the health achievements in quality-adjusted life-years (QALYs), incremental cost-effectiveness ratios (ICERs), and life-years (LYs). We constructed a treatment strategy and other parameters based on two clinical trials and performed one-way and probabilistic sensitivity experiments for the evaluation of the uncertainty in the model.

**Results:** For the model of patients with treatment-R/M NPC, TGP was associated with a total cost of $48,525 and 2.778 QALYs (4.991 LYs), leading to an ICER of $15,103 per QALY ($10,321 per LY) compared to CGP. On comparing the GP chemotherapy, we found TGP and CGP incurred substantial health costs, resulting in ICERs of $19,726 per QALY and $20,438 per QALY, respectively. The risk of adverse events (AEs) and the price of the drugs had significant impacts on the ICER. At the assumed willingness-to-pay (WTP) threshold of $35,673 per QALY, there were approximately 75.8 and 68.5% simulations in which cost-effectiveness was achieved for TGP and CGP, respectively.

**Conclusion:** From the Chinese payer’s perspective, TGP is more possible to be a cost-effective regimen compared with CGP and GP for first-line treatment of patients with R/M NPC at a WTP threshold of $35,673 per QALY.

## Introduction

Nasopharyngeal carcinoma (NPC) is a prevalent malignant tumor of the head and neck with high-incidence foci, and unique geographical distribution mainly distributed in southern China and Southeast Asia. According to the World Health Organization’s International Agency for Research on Cancer, 40% of global NPC cases occur in China (Torre et al., 2015). Approximately 10% of new cases are metastatic patients, while another 15–30% of locally advanced NPC patients will develop locally recurrent or disseminated disease following treatment for locally advanced NPC (Lee et al., 2015; Yang et al., 2015; Tan et al., 2016). Cases experiencing recurrent or metastatic nasopharyngeal carcinoma (R/M NPC) have a poor prognosis, with a median overall survival (OS) of only 20 months (Zhang et al., 2016).

Platinum-based regimens have been considered standard first-line chemotherapies for R/M NPC. In 2016, a phase 3 randomized controlled study (GEM20110714) confirmed that gemcitabine plus cisplatin (GP) was more efficient compared with fluorouracil plus cisplatin (PF) for the first-line treatment of R/M NPC [progression-free survival (PFS), hazard ratio (HR), 0.55; 95% confidence interval (CI), 0.44 to 0.68; *p* < 0.0001]. Although the adverse events (AEs) in the GP and PF groups were different, the overall safety was controllable. This trial was a milestone in determining the first-line treatment preference for R/M NPC. lthough its clinical benefit is limited, as the median PFS is only 7 months (Zhang et al., 2016). Thus, novel therapeutic strategies are essential for this group of patients.

Since the 21st century, immune checkpoint inhibitor (ICI) has gradually attracted the attention of tumor community (Glisch et al., 2020; Singh et al., 2020; Watson et al., 2020; Luo et al., 2021). And studies have increasingly confirmed the effectiveness of these immunotherapies for malignant tumors (Ferris et al., 2016; Burtness et al., 2019; Paz-Ares et al., 2019; Schmid et al., 2020; Colombo et al., 2021; Hua et al., 2021; Jiang et al., 2021; Paz-Ares et al., 2021; Zhou et al., 2021). Toripalimab and carrellizumab are humanized high-affinity PD-1 monoclonal antibodies showing good clinical effectiveness and safety as first-line therapies for R/M NPC. In September 2015, the Incyte Corporation reached an agreement with Hengrui Pharmaceutical Co. to purchase the overseas rights to carrellizumab for $795 million USD. Breakthrough therapy designation for Triplel was granted by the US Food and Drug Administration (FDA) for NPC treatment in September 2020. In JUPITER-02 (NCT03581786), a phase 3 study, toripalimab plus GP (TGP) significantly improved PFS compared with GP chemotherapy (median, 11.7 vs. 8.0 months; HR, 0.52; 95% CI, 0.36 to 0.74; *p* = 0.0003) (Mai et al., 2021). In CAPTAIN-first (NCT03707509), a phase 3 study, camrelizumab plus GP (CGP) extended PFS significantly compared with GP chemotherapy (median, 9.7 vs. 6.9 months; HR, 0.54; 95% CI, 0.39 to 0.76; *p* = 0.0002). Overall survival (OS) in both groups was immature, and preliminary data suggested that patients receiving carrilizumab combined with chemotherapy tended to have improved survival (median OS, NR vs. 22.6 months; HR, 0.67; 95% CI, 0.41–1.11) (Yang et al., 2021a). Based on these data, CGP and TGP were approved as first-line treatment options for cases experiencing R/M NPC by the National Medical Products Administration of China and the Chinese Society of Clinical Oncology and included in the protocols for NPC interventions, version2021^1^ ^,^ ^2^.

Although these treatment options have brought clinical benefits, the high cost and variety of ICIs means an analysis of their economics and efficacy is urgently essential to appraise which recently confirmed regimen presents the most clinical benefits at reasonable expenses and is more suitable for promotion. Therefore, the target of our study was to appraise the effectiveness and potential economic implications of TGP and CGP as first-line treatments for Chinese patients with R/M NPC from the Chinese citizen’s perspective.

## Materials and Methods

We conducted a network meta-analysis and cost-effectiveness analysis based on two phase 3 clinical trials. Details of the network meta-analysis methods are given in the Supplementary Material. Cost-effectiveness analysis is guided by the Economic Assessment Report Standard Statement (CHEERS) checklist (Supplementary Table S1) and the details of its methods are presented below.

### Model Structure

A Markov model with three exclusive health states was structured to demonstrate the possible consequences under evaluation: PFS, progressive disease (PD), and death (Supplementary Figure S2). Patients with R/M NPC were investigated and randomly assigned to receive one of three treatments in our study. In the PD state, patients deemed likely to gain clinical benefit received capecitabine (Martinez-Trufero et al., 2010), otherwise they were assigned to receive best supportive care (BSC) until death (National Comprehensive Ca, 2021).

The Markov cycle length was set at 6 weeks, with a 10-years horizon, based on the treatment regimen and expected survival time of R/M NPC patients. All costs, as well as health outcomes, were discounted by 3% annually (Ding et al., 2021). We chose the total expenses, quality-adjusted life-years (QALYs), life-years (LYs), and incremental cost-effectiveness ratios (ICERs) as primary endpoints with a willingness-to-pay threshold of $$35,673 per QALY (3 × capita gross domestic product of China in 2020) (Xiao et al., 2017). The simulation process was carried out on TreeAge Pro 2020 (TreeAge Computer program, Williamstown, MA, United States, https://www.treeage.com) and part of the statistical analysis was implemented in R (version 4.1.1, Available: http://www.rproject.org).

### Patients and Treatment

Based on two randomized controlled trials (RCTs) and the published literature, we hypothesized that patients with R/M NPC were male, 50 years old, 65 kg in weight, 164 cm in height, and had a body surface-area of 1.72 m^2^ (Liu et al., 2020; Yang et al., 2021a; Mai et al., 2021). We randomly assigned these patients to three groups: 1) a TGP group, which was treated with toripalimab (240 mg on day 1) plus gemcitabine (1,000 mg/m^2^ on days 1 and 8) and cisplatin (80 mg/m^2^ on day 1) every 3-weeks cycle for six cycles, succeeded by toripalimab only on day 1 of every 3-weeks cycle as maintenance (for a maximum of 2 years of treatment); 2) a CGP group, which was treated with camrelizumab (200 mg on day 1) plus gemcitabine (1,000 mg/m^2^ on days 1 and 8) and cisplatin (80 mg/m^2^ on day 1) every 3-weeks cycle for six cycles, succeeded by camrelizumab only on day 1 of every 3-weeks cycle as maintenance (for a maximum of 2 years of treatment); and 3) a GP group, which was treated with gemcitabine (1,000 mg/m^2^ on days 1 and 8) and cisplatin (80 mg/m^2^ on day 1) every 3-weeks cycle for six cycles, succeeded by placebo only on day 1 of every 3-weeks cycle as maintenance. Because, after disease development, follow-up treatment options for R/M NPC cases are generally restricted, and given that capecitabine chemotherapy was often used for follow-up treatment and specific drugs used for subsequent treatment were not specified in the JUPITER-02 and CAPTAIN-first clinical trials reports, we modeled that the patients received only capecitabine chemotherapy as follow-up treatment. Respectively, 32, 34, and 62% of patients in the TGP, CGP, and GP groups received subsequent chemotherapy. Treatment regimens and dosages were followed as detailed in the above clinical trials (Martinez-Trufero et al., 2010; Yang et al., 2021a; Mai et al., 2021). Specific usage details are listed in Supplementary Table S4.

### Model Survival and Transition Estimates

The survival outcomes were extracted from the curves of Kaplan-Meier (KM) of OS and PFS generated in the original JUPITER-02 and CAPTAIN-first trials using GetData Graph Digitizer (version 2.26; http://www.getdata-graph-digitizer.com/index.php). Based on these outcomes, transition probability (TP) between health states, death probability, and fitted long-term survival data were estimated. We evaluated five parametric survival models of fitness for the time-to-event data, including Weibull, Gompertz, exponential, log-logistic, and log-normal distributions. We selected the Weibull distribution as a survival model for PFS and PD according to the Bayesian information criterion and Akaike information criterion, the clinical rationality, and a visual inspection of the degree of similarity between the KM curves and 10-years extrapolated survival curves. The Surveillance, Epidemiology, and End Results data in the published literature indicated that log-normal and log-logistic distribution appeared not to fit with the reality of the long-term survival rate (Latimer, 2013). More details are shown in Supplementary Figure S4 and Supplementary Table S5. For the GP chemotherapy group, we used a network meta-approach to reconstruct the OS and PFS data at the individual patient level in both groups based on the JUPITER-02 and CAPTAIN-first trials. It is worth noting that we used the Weibull distribution for all treatment groups and obtained two parameters, scale (λ) and shape (γ), using the R software This study employed Hoyle’s suggested methodology (Hoyle and Henley, 2011) (Table 1).

**TABLE 1:** Model parameters: baseline values, ranges, and distributions for sensitivity analysis.

Parameters	Baseline Value	Range		Reference	Distribution
		Minimum	Maximum		
Survival					
Weibull survival model of OS of GP	Scale = 0.0004758	—	—	(6, 7)	—
Weibull survival model of PFS of GP	Shape = 2.3,014,344	—	—		—
	Scale = 0.011275				
	Shape = 1.991,263			
Weibull survival model of OS of TGP	Scale = 0.0016292	—	—	(6)	—
Weibull survival model of PFS of TGP	Shape = 1.6,248,844	—	—		—
	Scale = 0.010542				
	Shape = 1.663,938				
Weibull survival model of OS of CGP	Scale = 0.005613	—	—	(7)	—
Weibull survival model of PFS of CGP	Shape = 1.319,492	—	—		—
	Scale = 0.011551				
	Shape = 1.762,665				
Risk for main AEs in GP group					
Risk of neutropenia	0.471	0.377	0.565	(6, 7)	Beta
Risk of anemia	0.412	0.330	0.494	(6, 7)	Beta
Risk of thrombocytopenia	0.342	0.274	0.410	(6, 7)	Beta
Risk of leucopenia	0.636	0.509	0.763	(6, 7)	Beta
Risk of lymphopenia	0.129	0.103	0.155	(6, 7)	Beta
Risk for main AEs in TGP group					
Risk of leucopenia	0.616	0.493	0.739	(6)	Beta
Risk of neutropenia	0.575	0.460	0.690	(6)	Beta
Risk of anemia	0.473	0.378	0.568	(6)	Beta
Risk of thrombocytopenia	0.329	0.263	0.395	(6)	Beta
Risk of lymphopenia	0.089	0.071	0.107	(6)	Beta
Risk of hyponatremia	0.089	0.071	0.107	(6)	Beta
Risk of hypokalemia	0.068	0.054	0.082	(6)	Beta
Risk of pneumonia	0.103	0.082	0.124	(6)	Beta
Risk for main AEs in CGP group					
Risk of neutropenia	0.24	0.19	0.29	(7)	Beta
Risk of anemia	0.09	0.07	0.11	(7)	Beta
Risk of thrombocytopenia	0.06	0.05	0.07	(7)	Beta
Risk of leucopenia	0.06	0.05	0.07	(7)	Beta
Risk of neutrophil count decreased	0.06	0.05	0.07	(7)	Beta
Risk of febrile neutropenia	0.06	0.05	0.07	(7)	Beta
Risk of hyponatraemia	0.06	0.05	0.07	(7)	Beta
Utility and disutility					
Utility PFS in first-line treatment	0.65	0.520	0.780	(16)	Beta
Utility PD	0.52	0.416	0.624	(16)	Beta
AEs disutility for GP	0.0069	0.0055	0.0083	(17)	Beta
AEs disutility for TGP or CGP	0.0070	0.0056	0.0084	(17)	Beta
Drug cost, $/per cycle					
Toripalimab	659.4	527.52	791.28	Local Charge	Gamma
Camrelizumab	888.3	710.64	1,065.96	Local Charge	Gamma
Gemcitabine	860.9	688.72	1,033.08	Local Charge	Gamma
Cisplatin	332.1	265.68	398.52	Local Charge	Gamma
Capecitabine	128.0	102.40	153.60	Local Charge	Gamma
Cost of AEs, $					
GP	1,940	1,552	2,328	(18, 21–23)	Gamma
TGP	1,980	1,584	2,367	(18, 21–23)	Gamma
CGP	2,246	1,797	2,695	(18, 21–23)	Gamma
Laboratory per cycle	216.4	173.12	259.68	(19)	Gamma
Tumor imaging per cycle	231.1	184.80	277.20	(19)	Gamma
Administration per cycle	106.2	84.96	127.44	(18)	Gamma
Best supportive care per cycle	157.6	126.08	189.12	(20)	Gamma
Body surface area (meters2)	1.72	1.38	2.06	(13)	Gamma
Discount rate	0.03	—	—	(11)	—

Abbreviation; OS, overall survival; PFS, progression-free survival; GP, gemcitabine and cisplatin; TGP, toripalimab plus gemcitabine and cisplatin; CGP, camrelizumab plus gemcitabine and cisplatin; AEs, adverse events.

### Utilities and Cost Inputs

Utility was used to reflect the weight of the patients’ quality of life in the natural background of the disease on a scale of 0 (death) to 1 (total health). We regarded utility scores of 0.65 and 0.52 for PFS state and PD state, accordingly, as published by Jin et al. (2020). We assessed the impact of the deterioration of the quality of life contingent on clinical events as the disutility multiplied by the incidence of severe AEs (Tringale et al., 2018) (Table 1).

We only considered direct costs from the Chinese social perspective and converted to US dollars as of the 2021 conversion rate. The Chinese Yuan was converted into USD using the following exchange formula: 1US $ = CNY 6.4. The costs were calculated for medicines, administration (Lang et al., 2020), tumor imaging (Yang et al., 2020), laboratory tests (Yang et al., 2020), BSC (Xin et al., 2020), and management of severe AEs (assuming that Aes emerged only once in the PFS and PD states) (Guan et al., 2019; Lang and Dong, 2020; Lang et al., 2020; Li et al., 2020) (Table 1). Grades 3 to 4 Aes with an incidence rate of ≥5% in either group or with significantly different rates between groups were estimated in the calculation. In addition, due to the different reimbursement rates of medical insurance in different regions of China, we excluded preferential policies in the cost input.

### Sensitivity Analysis

We executed univariable sensitivity assessments to indicate the uncertainty and impact of the parameters among the treatment alternatives using the available evidence. Univariable sensitivity analysis evaluated specific parameters in JUPITER-02 and CAPTAIN-first trials and 20% variation from baseline values (Ding et al., 2021). Probabilistic sensitivity analysis was performed to characterize the current decision uncertainties. A Monte Carlo simulation was conducted 10,000 times employing scatterplot and acceptability curves on the cost-effectiveness plane to examine the probability of being cost-effective.

We pooled the HR and 95% CI for the OS and PFS of each treatment group in the two RCTs based on indirect comparisons and used R computer program (version 4.1.1, http://www.r-project.org) for comparative analysis. However, as only one RCT involved a pairwise comparison of individuals, and due to the lack of a dataset to assess heterogeneity across the trials, we developed a fixed-effect model (Rücker and Schwarzer, 2015). Therefore, the frequency method was employed for the comparison of the comparative effectiveness of various schemes. The HR of OS and PFS, and the corresponding 95% Cis and *p*-values, were evaluated, and the *p*-value of each result was used for ranking, where a higher value indicated higher success.

## Results

### Network Meta-Analysis

A database search identified 187 records through a database search, and two phase III randomized clinical trials (JUPITER-02 and CAPTAIN-first) involving 552 patients were included in the meta-analysis (Supplementary Figure S4 and Supplementary Table S3). In examining the JUPITER-02 trial, 289 patients received TGP or GP; In the JUPITER-02 trial, 263 patients received either CGP or GP treatment. The risk of bias is shown in Supplementary Figure S5.

### Baseline Results

For patients with R/M NPC with a 10-years horizon, TGP presented an additional 0.148 QALYs (0.240 Lys) at an increased cost of $2,232 compared with CGP, leading to an ICER value of $15,103 per QALY ($10,321 per LY). A comparison of the two chemo-immunotherapies and GP chemotherapy showed that the addition of toripalimab and camrelizumab to first-line GP chemotherapy yielded 1.108 and 0.960 QALYs (2.015 and 1.799 Lys), respectively. Due to the QALY improvement, TGP and CGP involve higher medical costs than GP chemotherapy, resulting in ICERs of $19,726 and $20,438 per QALY ($10,842 and $10,904 per LY), respectively (Table 2).

**TABLE 2:** Baseline results.

Parameters	TGP	CGP	GP
LYs	4.991	4.751	2.976
QALYs	2.778	2.630	1.670
Total cost $	48,525	46,293	26,680
ICER $/LY	10,842^a^	10,904^a^	—
	10,321^b^		
ICER $/QALY	19,726^a^	20,438^a^	—
	15,103^b^		
WTP $/QALY	37,653		

a Compared to GP.

b Compared to CGP.

Abbreviation: TGP, toripalimab plus gemcitabine and cisplatin; CGP, camrelizumab plus gemcitabine and cisplatin; GP, gemcitabine and cisplatin; ICER, incremental cost-effectiveness ratio; LY, life-year; QALY, quality-adjusted life-year; WTP, willingness-to-pay.

### Sensitivity Analysis Results

**FIGURE 1:**
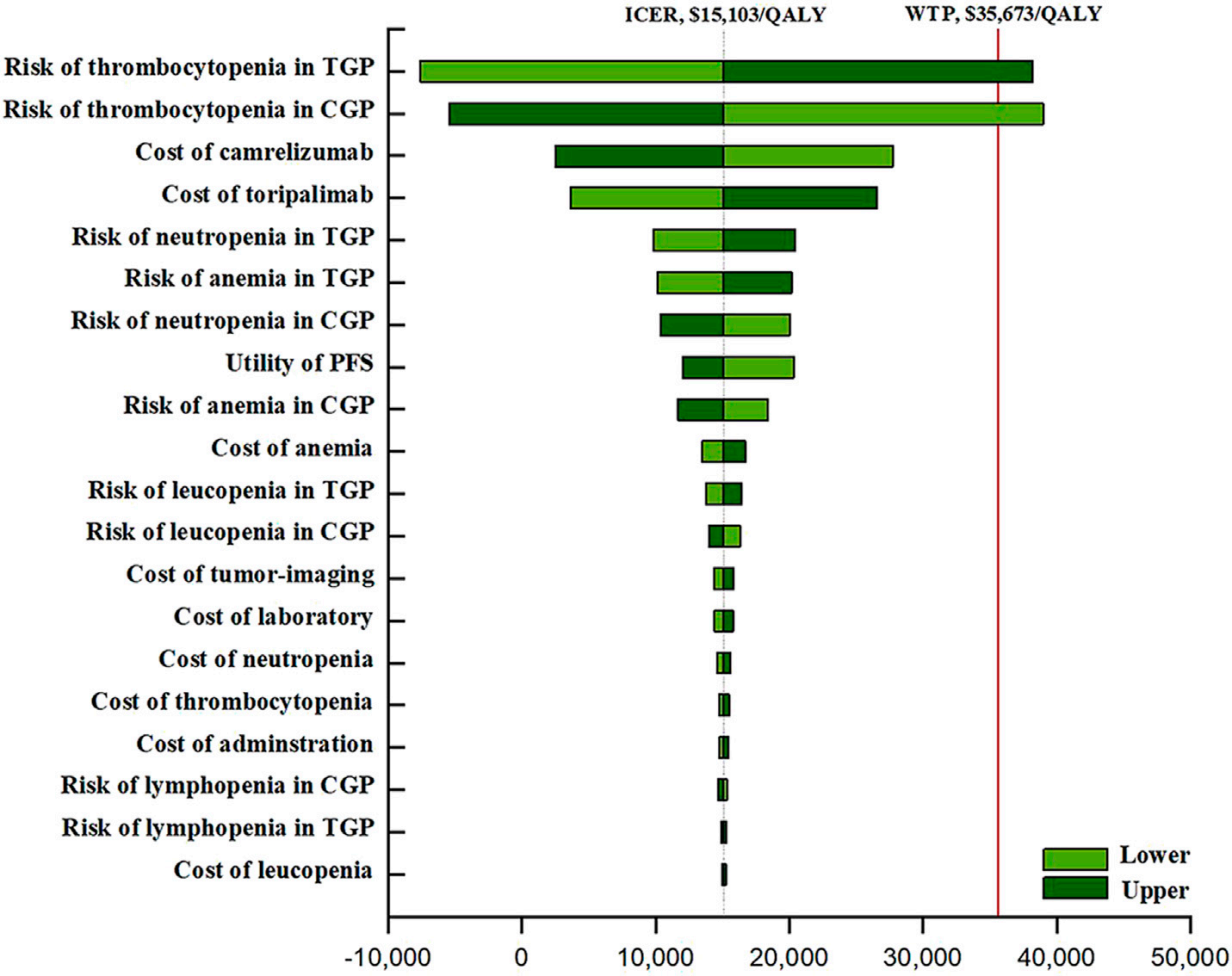
The one-way sensitivity analyses. Abbreviation: TGP, toripalimab plus gemcitabine and cisplatin; CGP, camrelizumab plus gemcitabine and cisplatin; GP, gemcitabine plus cisplatin; PFS, progression-free survival; BSC, best supportive care.

The outcomes of the one-way sensitivity assessment showed a high sensitivity to the risk of thrombocytopenia for TGP (ranging from 26.3 to 39.5%, with the ICER raising from -$7,598 per QALY to $38,195 per QALY). Other significant influencing factors such as the cost of the ICI, the utility of the PFS, and the incidences of anemia and neutropenia. Other factors considered in the analysis of sensitivity, for instance, the cost of chemotherapy drugs and Aes, had little impact on the ICER (Figure 1).

**FIGURE 2:**
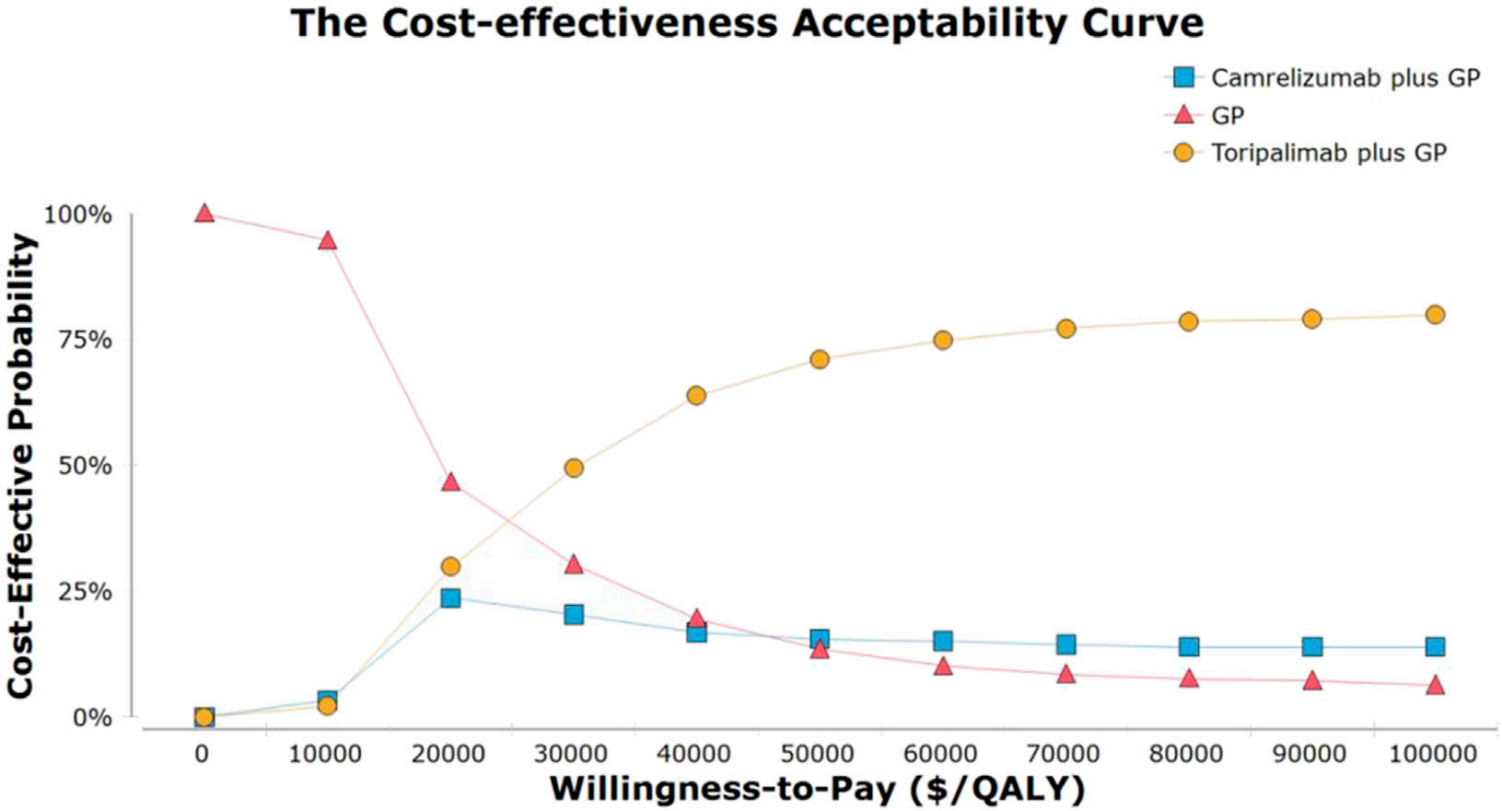
The cost-effectiveness acceptability curves for the toripalimab plus gemcitabine and cisplatin, camrelizumab plus gemcitabine and cisplatin. Abbreviation: GP, gemcitabine plus cisplatin.

As demonstrated in the curve of cost-efficiency acceptability, the probability that the strategy of TGP is cost-efficient increased as the WTP for additional QALY rose (Figure 2). GP chemotherapy was the optimal strategy when WTP was less than$30,000/QALY. When WTP was greater than or equal to, $30,000/QALY TGP was found to be the optimal strategy. The scatter plots demonstrated that, at a WTP threshold of $35,673 per QALY, the TGP and CGP strategies were cost-effective in 75.8 and 68.5% of the simulations (Supplementary Figure S6).

**FIGURE 3:**
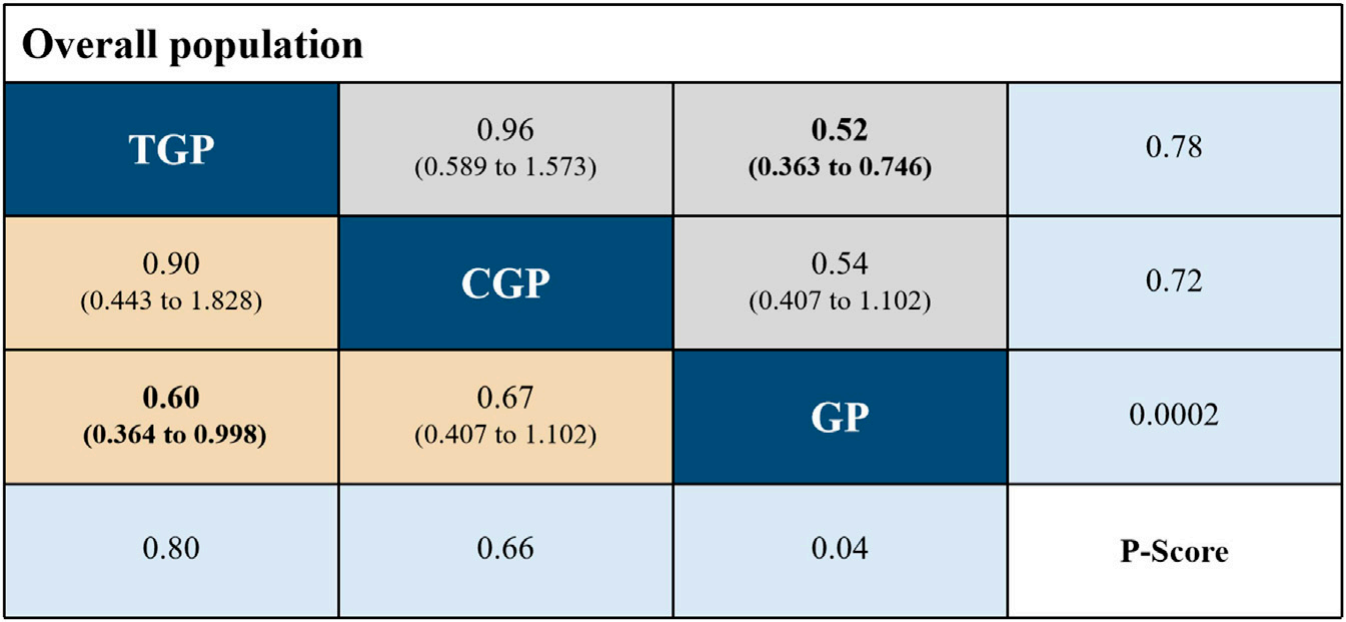
The pooled HR; 95%CI and *p*-values for OS (lower triangle) and PFS (upper triangle) of the network meta-analysis; significant results are in bold. Abbreviation: TGP, toripalimab plus gemcitabine and cisplatin; CGP, camrelizumab plus gemcitabine and cisplatin; GP, gemcitabine plus cisplatin; OS, overall survival; PFS, progression-free survival.

An indirect comparison of the data revealed that TGP (HR, 0.60; 95% CI, 0.364–0.998 and HR, 0.52; 95% CI, 0.363–0.746) led to meaningful statistical enhancements in OS and PFS in comparison to GP. No statistically meaningful discrepancies in OS and PFS were detected across the two chemo-immunotherapy regimens. The best treatment achievements were indicated by the *p*-values (for individual outcomes), where higher values indicated the treatment was more successful. Among the overall populations, the regimen with peak *p*-values for OS and PFS was TGP (*p* = 0.80 and *p* = 0.78), followed by CGP (*p* = 0.66 and *p* = 0.72), and GP (*p* = 0.04 and *p* = 0.0002), respectively. The findings of the indirect comparisons and the *p*-values for the OS and PFS of each regimen are illustrated in Figure 3.

## Discussion

Over the past decade, immunotherapy has become one of the most important breakthroughs in cancer treatment (Sharon et al., 2014). PD-1 blocking antibody has good therapeutic effects on recurrent or metastatic head and neck cancer (R/M HNC), but there have been few corresponding economic evaluations of R/M HNC. The cost-effectiveness of pembrolizumab for R/M HNC patients demonstrated its cost-effectiveness compared to standard treatments in both China, the United States, and Argentina (Liu et al., 2019; Wurcel et al., 2021). A cost-effectiveness study by Robert et al. demonstrated that nivolumab was more cost-effective compared to chemotherapy in cases experiencing R/M HNSCC in the US (Haddad et al., 2020). In addition, network meta-analysis and cost-effectiveness analysis of R/M HNSCC patients in the US found that using nivolumab or pembrolizumab was more cost-effective based on WTP thresholds and patient weight (Pei et al., 2021). However, NPC is a type of HNC, and so far, there have been no cost-effectiveness analyses of immunotherapy for these patients; thus, a cost-effectiveness analysis of R/M NPC is crucial.

Individuals undergoing cancer treatment face a wide range of treatment options, but they and the societies they belong to face increasing economic burdens due to limited medical resources and high treatment prices. Economic assessment is a direct and theory-based approach that measures both the costs and outcomes in view of individual and social choices. Using the Markov model, we executed the first cost-effectiveness assessment of R/M NPC treatments from the Chinese citizens perspective and assessed the cost and efficacy of TGP, CGP, and GP chemotherapies at a WTP of $35,673 per QALY. Through our study, we found that TGP and CGP treatments resulted in an additional cost of $21,846 and $19,613, resulting in 1.108 and 0.960 QALYs compared to GP, respectively, which are clearly below the threshold for WTP. And ICER was significantly lower than the threshold of WTP. In addition, after comparing the two schemes, the ICER for TGP was deemed to be $15,103/QALY. Therefore, this cost-effectiveness study conducted for cases with R/M NPC in China demonstrated that TGP is the more efficient first-line treatment strategy and achieves the highest cost-effectiveness compared to CGP and GP.

The robustness of the model was verified through a sensitivity analysis of the model parameters. The most influential parameters in this model were the risk of thrombocytopenia from TGP and CGP, followed by the cost of toripalimab and camrelizumab. We found that the incidence of thrombocytopenia from TGP decreased by more than 15%, and the price of toripalimab decreased by more than 30% while that of camrelizumab rose by more than 30%, allowing TGP to dominate CGP economically. Because changing other parameters had no substantial effect on our results, mitigating drug-induced AEs and reducing the price of ICIs were considered the most practical measures for first-line TGP and CGP treatment to become absolutely cost-effective. It is worth noting that real-world evidence and many clinical studies have shown that grade 3/4 thrombocytopenia in chemo-immunotherapy is most likely to be caused by the chemotherapy. The incidences of grade 3/4 thrombocytopenia in NPC patients treated with GP chemotherapy alone and immunotherapy alone were over 10% and almost 0%, respectively (Zhang et al., 2016; Zhang et al., 2019; Yang et al., 2021a; Yang et al., 2021b; Mai et al., 2021). In clinical practice, if severe thrombocytopenia occurs during the treatment of NPC patients given chemo-immunotherapy, we first adjust the dose of chemotherapy to reduce its incidence, then reduce the ICER of the treatment regimen.

For a new drug to be approved by government and used correctly and widely in clinical situations, we need to rely on using clinical survival benefits, regional economic factors, and predictive markers to make judgements. For example, plasma Epstein-Barr virus deoxyribonucleic acid (EBV-DNA) levels have a substantial prognostic influence in cases with NPC. A retrospective analysis of 210 patients with NPC revealed a worse relapse-free survival rate (79.3%; *p* < 0.0001) and poorer OS (86%; *p* = 0.0003) in cases who were given high pretreatment EBV-DNA levels (Wang et al., 2013). A meta-analysis involving 22 investigations and 8,128 NPC cases showed that patients with high levels of EBV-DNA had a five to six times higher risk of death and metastasis than patients with low levels (Qu et al., 2020). However, programmed cell death ligand-1 **(**PD-L1) expression confirmed to be a proper biomarker for predicting the clinical effectiveness and prognosis of ICIs in HNC (Li et al., 2017; Cao et al., 2019; Huang et al., 2021). Li and others’ retrospective analysis proved that the 5-years OS and PFS of 120 nasopharyngeal carcinoma patients were 87.5 and 70.1%, respectively (Li et al., 2017). Another retrospective analysis showed a PD-L1 expression of 0%, 1–5%, 5–49%, and ≥50% in 154 NPC patients and 5-years OS and PFS of 75.5 and 85.7%, 72.7 and 72.7%, 55.9 and 68.3%, and 24.8 and 35%, respectively (Cao et al., 2019). Another meta-analysis suggested that high or positive expression of PD-L1 in head and neck squamous cell carcinoma (HNSCC) has a good predictive effect for OS at 6 and 12 months [relative risk (RR), 1.30; 95% CI, 1.02 to 1.65; *p* = 0.03; RR, 1.31; 95% CI, 1.05 to 1.62; *p* = 0.01] (Huang et al., 2021). In addition to the above two main biomarkers of clinical efficacy and prognosis, many studies have also indicated that the expression of epidermal growth factor receptor, Ki-67, vascular endothelial growth factor, and BRAF may also be good predicting biomarkers for NPC patients (Cheng et al., 2018; Cao et al., 2019; Chen et al., 2020; Li et al., 2021). Unfortunately, the JUPITER-02 and CAPTAIN-first studies lacked OS data for these two major prognostic markers, and PD-L1 expression was not grouped in CAPTAIN-first, hence, these could not be analyzed. The biomarkers that might lead specific patients to benefit from immuno-chemotherapy need to be confirmed through further research, which may make personalized treatment possible.

Some limitations were also evident in this study. First, an indirect comparison between first-line TGP and CGP was performed using network meta-analysis. Because we assumed no difference in patient characteristics between the two studies, there is potential uncertainty regarding the accuracy. Second, due to the short follow-up period of the two clinical trials, it was necessary to extrapolate the survival curve to obtain complete survival outcomes. The survival data will change over time, and the model will become more stable as more mature data becomes available. However, for now, this is an unavoidable limitation in our model. Third, to simplify the calculation, we assumed that follow-up treatment in the three groups only involved capecitabine chemotherapy, with the highest probability in the two studies, and ignored the other treatment options. On this basis, the analysis may have underestimated the cost of PD. However, sensitivity assessment demonstrated that changing the cost of capecitabine had little effect on the modelled results. Finally, considering that immunotherapy-related AEs are rare (the incidence was less than 10% in the two studies) and the cost of their treatment is quite high, we overlooked their administrative costs, which may overestimate the benefit of chemo-immunotherapy. However, including the cost of AE cases associated with immunotherapy will help to more accurately assess the overall cost of treating AEs using chemo-immunotherapy.

Conclusively, our achievements explain that TGP regimens could be more cost-efficient than GP and CGP regimens in China at a WTP threshold of $35,673 per QALY. It is necessary to provide patients the most efficacious treatment at the lowest cost, and the findings may help clinicians select the most appropriate drugs for patients and develop policies for medical reimbursement.

## Data Availability

The original contributions presented in the study are included in the article/Supplementary Material, further inquiries can be directed to the corresponding author.

## Footnotes

1
http://www.csco.org.cn/cn/index.aspx Accessed, 2021.

2
https://www.nmpa.gov.cn Accessed, 2021.
