# Supplementary material for: Chemo-Immunotherapy Regimes for Recurrent or Metastatic Nasopharyngeal Carcinoma: A Network Meta-Analysis and Cost-Effectiveness Analysis
Source: Front Pharmacol. 2022 May 20;13:858207. doi: 10.3389/fphar.2022.858207 (PMC9163401; doi:10.3389/fphar.2022.858207)
Supplement: Supplementary file 1 [file DataSheet1.PDF]

eFigure 1. Study Selection

eFigure 2. Model structure for cost-effectiveness analysis.

eFigure 3. Kaplan-Meier Curve Fitting and Extrapolation.

eFigure 4. Model of Network Meta-analysis.

eFigure 5. Risk of Bias Summary.

eFigure 6. Probability Sensitivity Analysis Scatter Plot.

eTable 1. CHEERS Checklist.

eTable 2. Search strategy.

eTable 3. Characteristics of RCTs included in the study.

eTable 4. Drug dose and cost.

eTable 5. Summary of statistical goodness-of-fit of K-M curve.

eMethods. Network Meta-analysis.

eReferences.

eFigure 1. Study Selection.

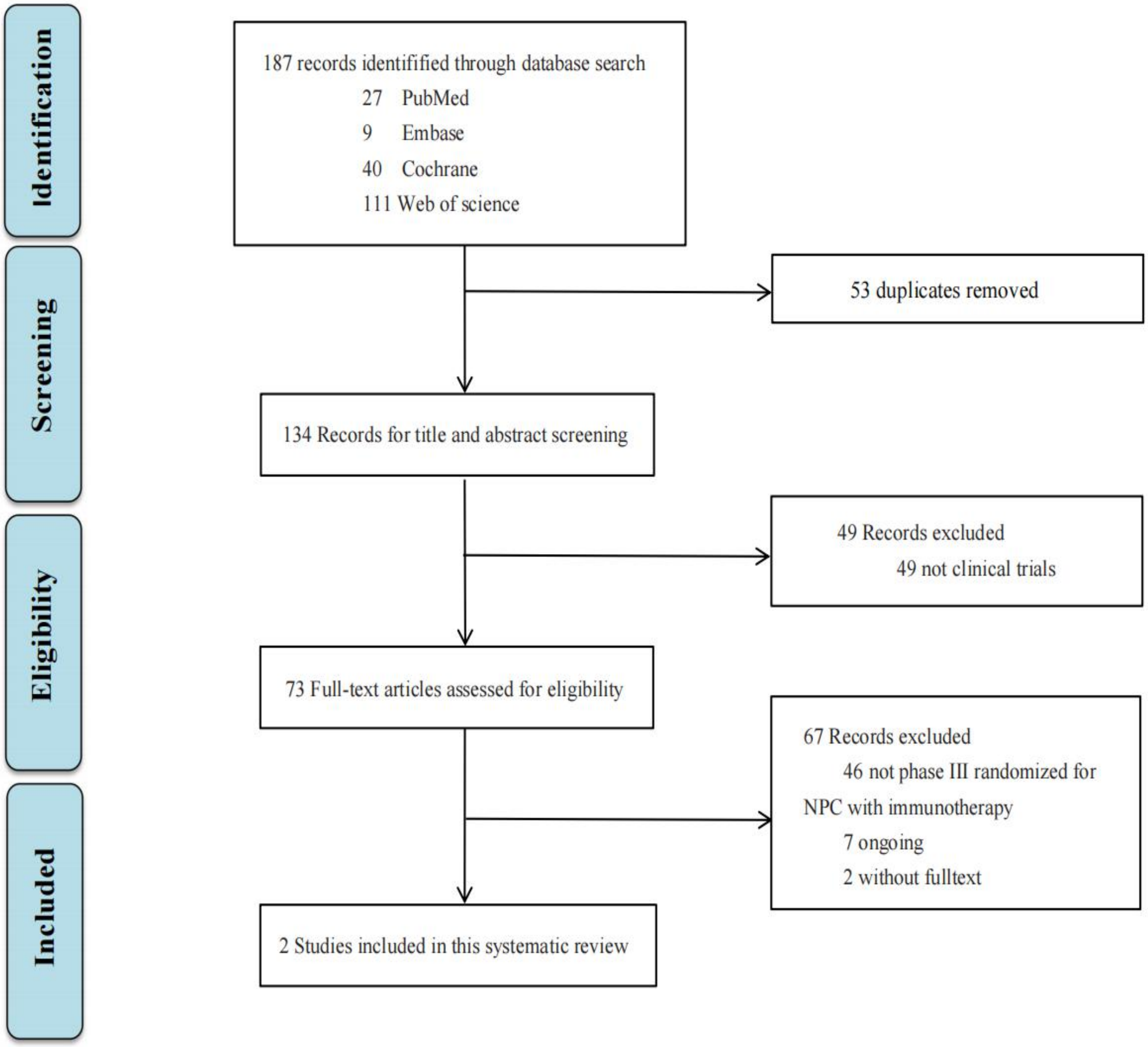

\

eFigure 2. Model Structure for Cost-effectiveness Analysis.

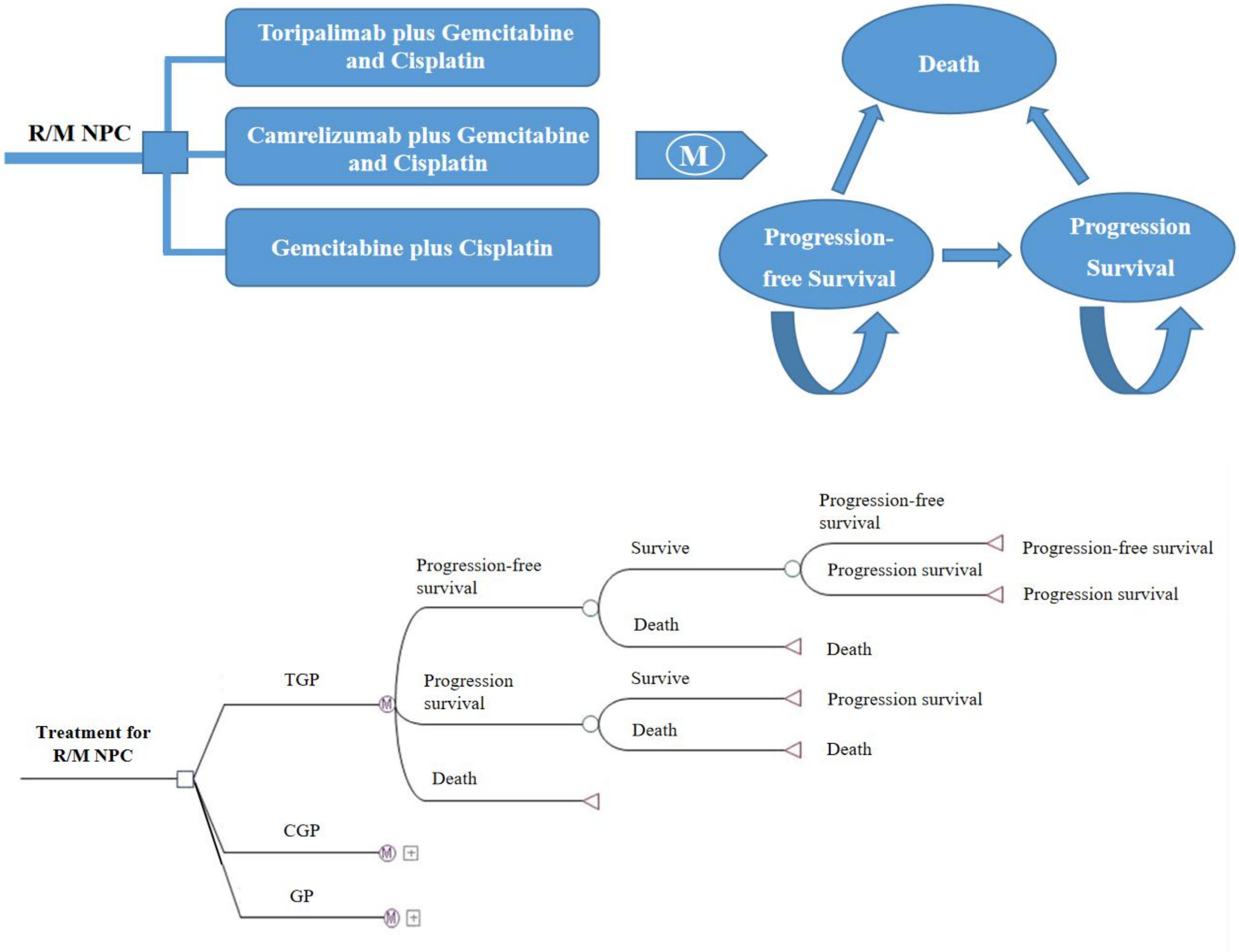

Abbreviation: R/M NPC, Recurrent or metastatic nasopharyngeal carcinoma; TGP, toripalimab plus gemcitabine and cisplatin; CGP, camrelizumab plus gemcitabine and cisplatin; GP, gemcitabine plus cisplatin; M, Markov.

eFigure 3. Kaplan-Meier Curve Fitting and Extrapolation.

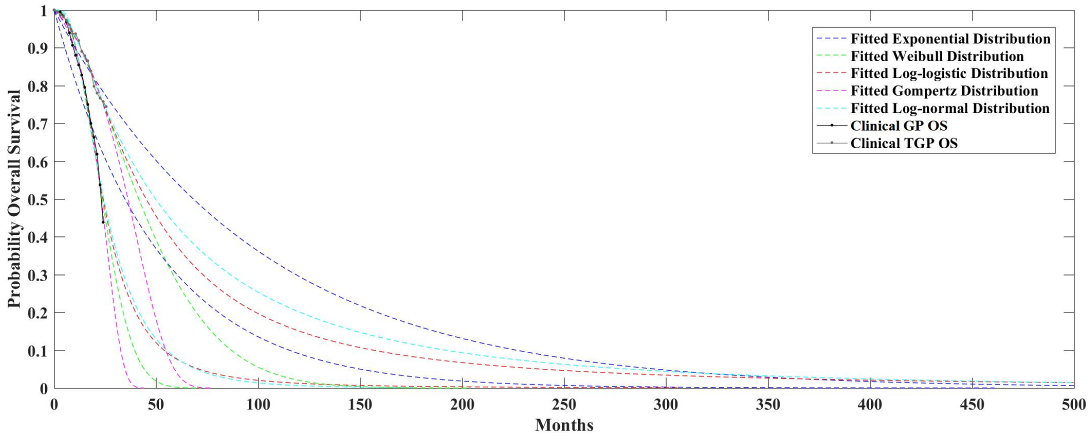

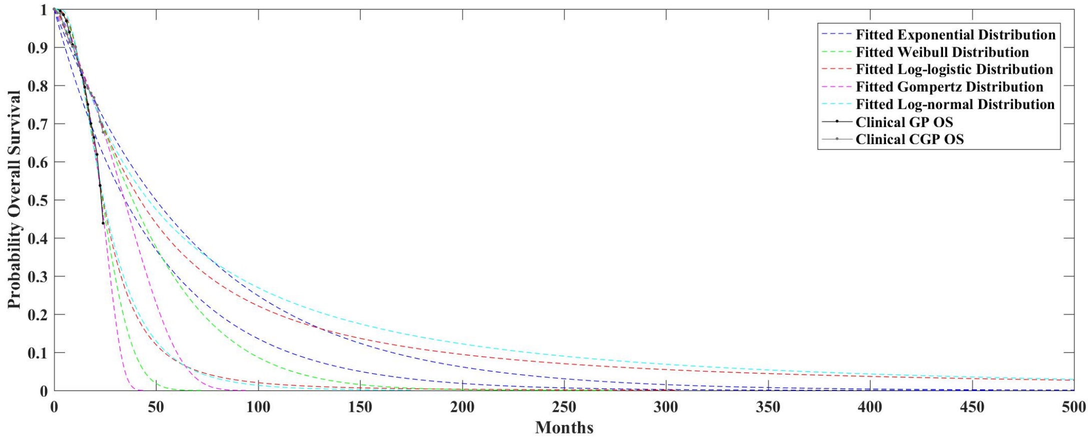

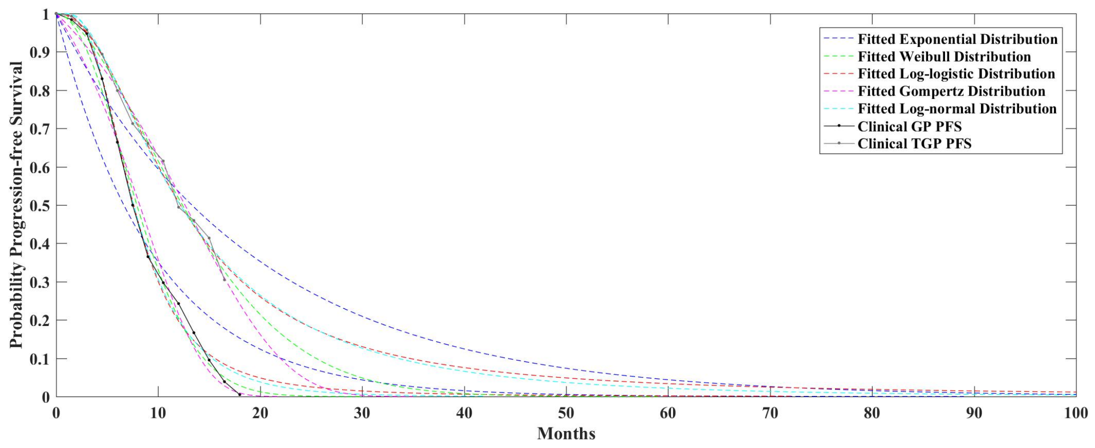

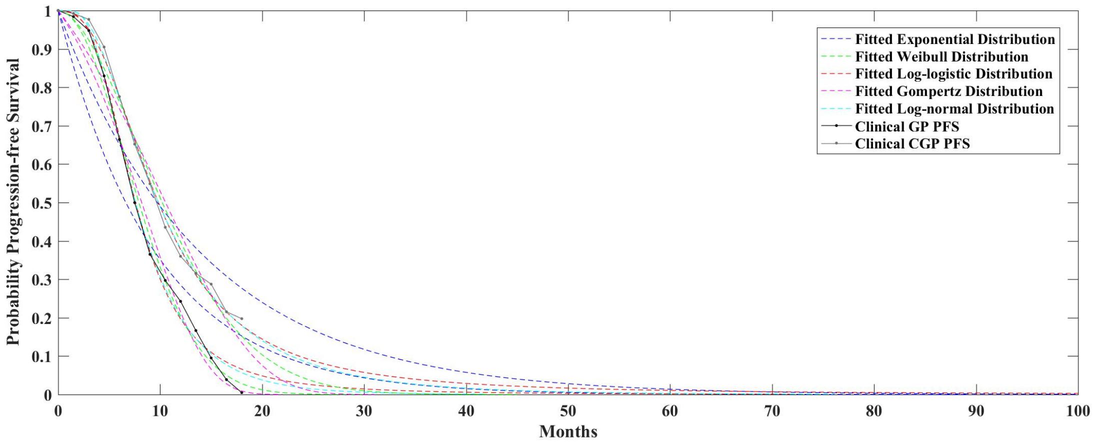

Abbreviation: TGP, toripalimab plus gemcitabine and cisplatin; CGP, camrelizumab plus gemcitabine and cisplatin; GP, gemcitabine plus cisplatin; OS, overall survival; PFS, progression-free survival

eFigure 4. Model of Network Meta-analysis.

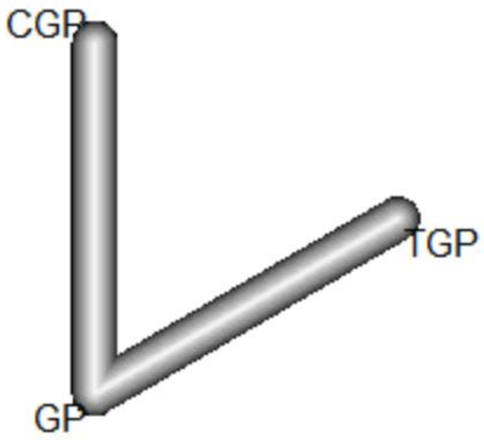

Abbreviation: TGP, toripalimab plus gemcitabine and cisplatin; CGP, camrelizumab plus gemcitabine and cisplatin; GP, gemcitabine plus cisplatin

eFigure 5. Risk of Bias Summary.

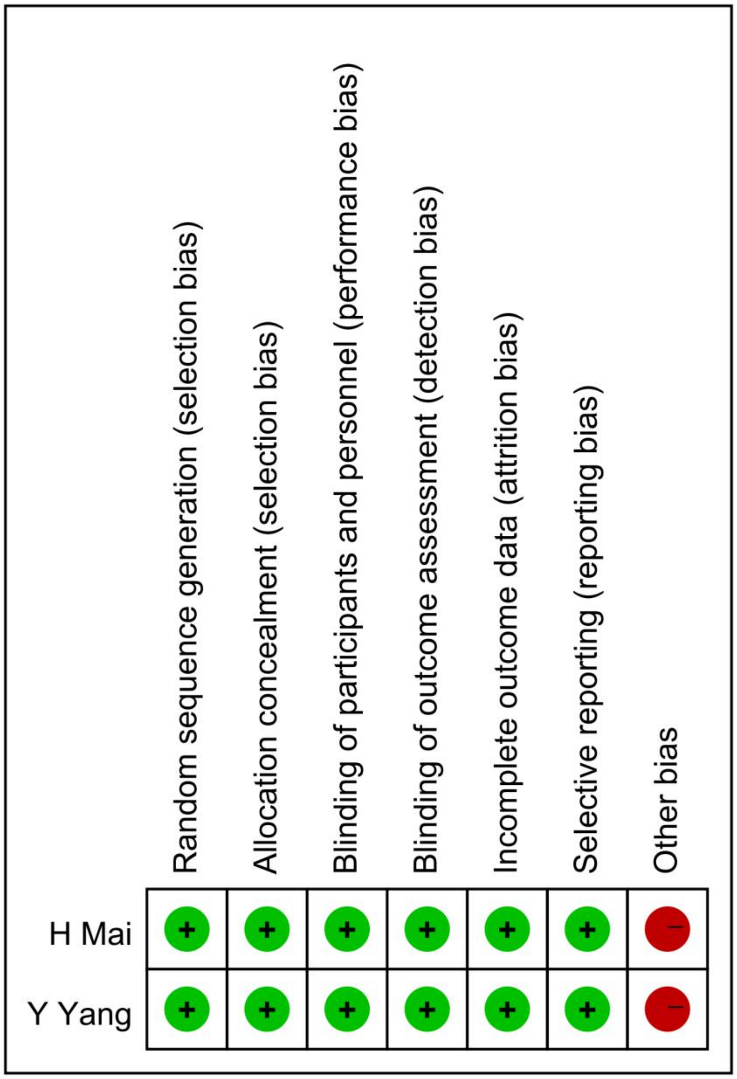

The CAPTAIN-1st and JUPITER-02 was judged at low risk in random sequence generation and allocation concealment due to the using of computerized randomization and a permuted block of flexible size. In the CAPTAIN-1st and JUPITER-02, given patients investigators were masked to group assignment, the performance bias was categorized as at low risk, while the outcome was assessed by masked, independent central review, so the detection bias was considered as at low risk. Two studies were considered at low risk of bias for incomplete outcome data. They were at low risk in the reporting bias given both had research proposals and reported all the predetermined outcome indicators (primary and secondary outcomes). Regarding other bias, the results may be skewed to the better because the main treatment drug is sponsored by corporations, we attributed them to high risk.

eFigure 6. Probability Sensitivity Analysis Scatter Plot.

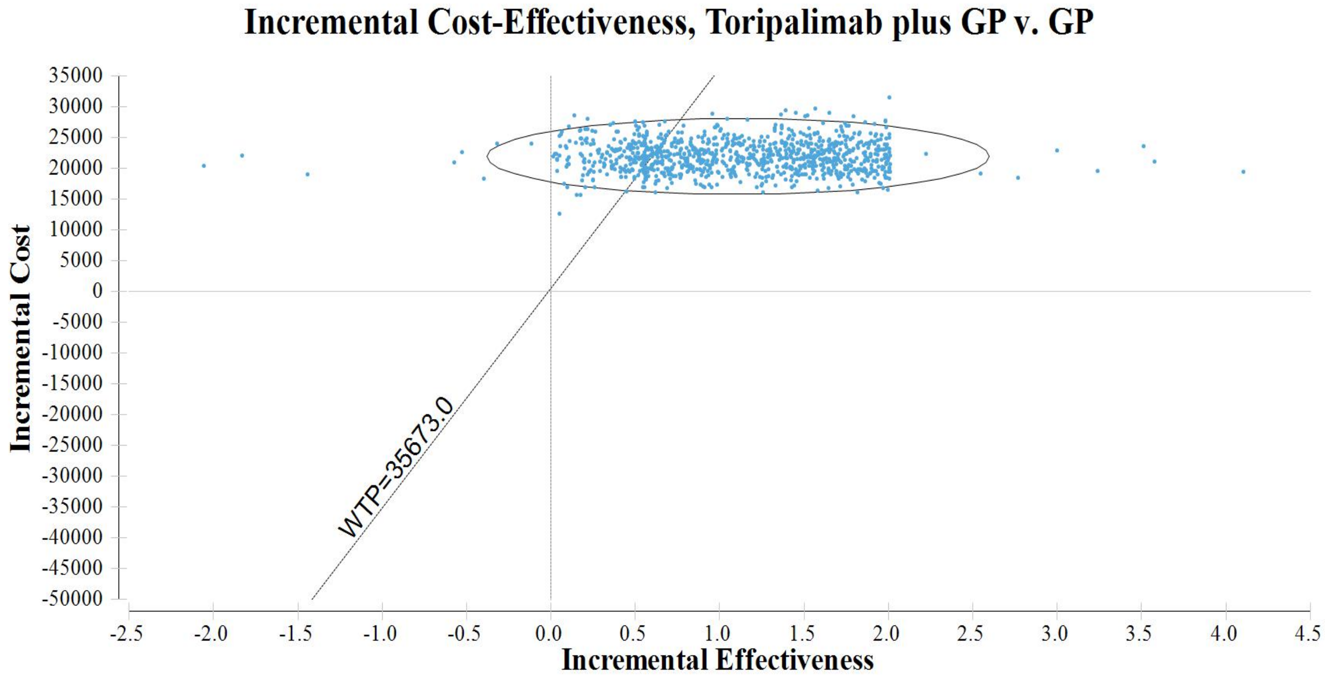

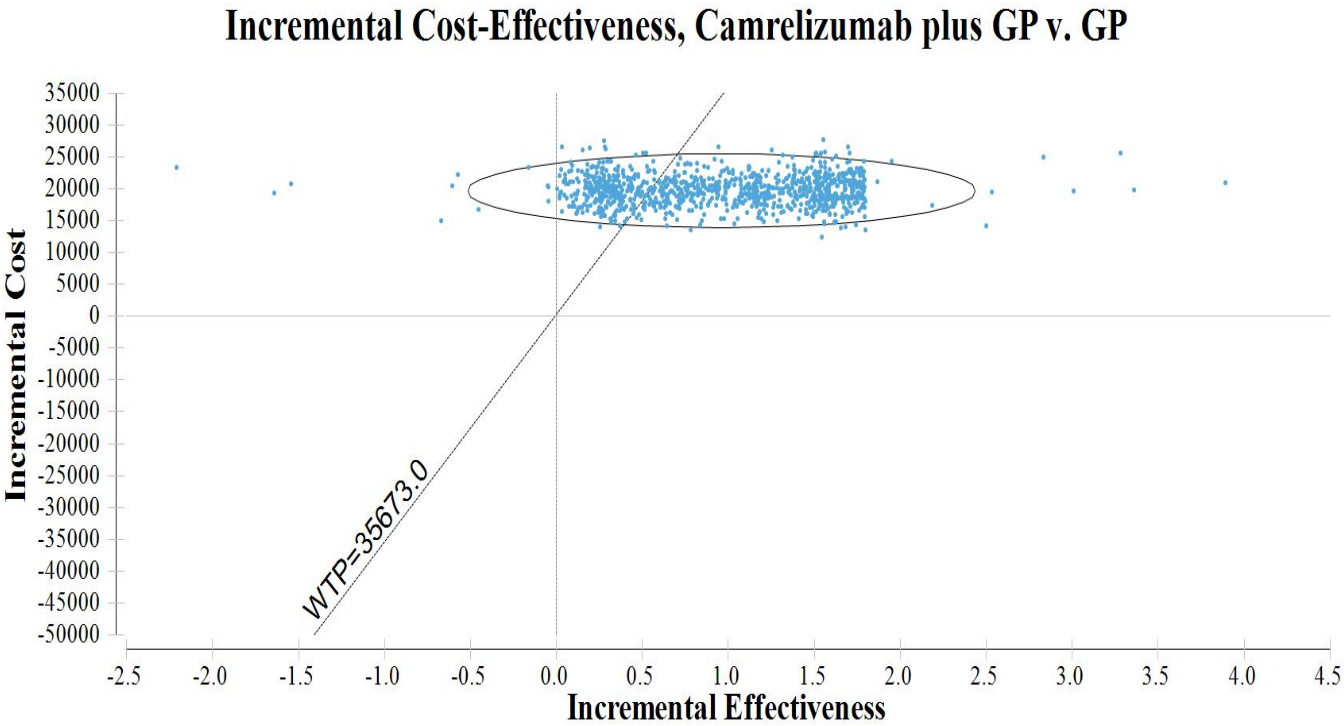

Abbreviation: GP, gemcitabine plus cisplatin; WTP, willingness-to-pay.

Each point in the diagram represents a simulation result of 10,000 Monte Carlo simulation. Ellipse represent the 95% CI and dotted line represent WTP ($35,673/QALY). Points to the right of the dotted line are considered cost-effective.

eTable 1. CHEERS Checklist.

Section/item	Item No	Recommendation	Reported on page No
**Title and abstract**			
Title	1	Identify the study as an economic evaluation or use more specific terms such as "cost-effectiveness analysis", and describe the interventions compared.	1
Abstract	2	Provide a structured summary of objectives, perspective, setting, methods (including study design and inputs), results (including base case and uncertainty analyses), and conclusions.	2
**Introduction**			
Background and objectives	3	Provide an explicit statement of the broader context for the study.	3-4
		Present the study question and its relevance for health policy or practice decisions.	
**Methods**			
Target population and subgroups	4	Describe characteristics of the base case population and subgroups analysed, including why they were chosen.	7
Setting and location	5	State relevant aspects of the system(s) in which the decision(s) need(s) to be made.	7
Study perspective	6	Describe the perspective of the study and relate this to the costs being evaluated.	7
Comparators	7	Describe the interventions or strategies being compared and state why they were chosen.	7
Time horizon	8	State the time horizon(s) over which costs and consequences are being evaluated and say why appropriate.	7
Discount rate	9	Report the choice of discount rate(s) used for costs and outcomes and say why appropriate.	7
Choice of health outcomes	10	Describe what outcomes were used as the measure(s) of benefit in the evaluation and their relevance for the type of analysis performed.	7
Measurement of effectiveness	11a	*Single study-based estimates:* Describe fully the design features of the single effectiveness study and why the single study was a sufficient source of clinical effectiveness data.	6

	11b	*Synthesis-based estimates:* Describe fully the methods used for identification of included studies and synthesis of clinical effectiveness data.	
Measurement and valuation of preference based outcomes	12	If applicable, describe the population and methods used to elicit preferences for outcomes.	7-8
Estimating resources and costs	13a	*Single study-based economic evaluation:* Describe approaches used to estimate resource use associated with the alternative interventions. Describe primary or secondary research methods for valuing each resource item in terms of its unit cost. Describe any adjustments made to approximate to opportunity costs.	8-9
	13b	*Model-based economic evaluation:* Describe approaches and data sources used to estimate resource use associated with model health states. Describe primary or secondary research methods for valuing each resource item in terms of its unit cost. Describe any adjustments made to approximate to opportunity costs.	
Currency, price date, and conversion	14	Report the dates of the estimated resource quantities and unit costs. Describe methods for adjusting estimated unit costs to the year of reported costs if necessary. Describe methods for converting costs into a common currency base and the exchange rate.	8
Choice of model	15	Describe and give reasons for the specific type of decision-analytical model used. Providing a figure to show model structure is strongly recommended.	8 and eFigure 5
Assumptions	16	Describe all structural or other assumptions underpinning the decision-analytical model.	8
Analytical methods	17	Describe all analytical methods supporting the evaluation. This could include methods for dealing with skewed, missing, or censored data; extrapolation methods; methods for pooling data; approaches to validate or make adjustments (such as half cycle corrections) to a model; and methods for handling population heterogeneity and uncertainty.	8-9
**Results**			

Study parameters	18	Report the values, ranges, references, and, if used, probability distributions for all parameters. Report reasons or sources for distributions used to represent uncertainty where appropriate. Providing a table to show the input values is strongly recommended.	8 (Table 1)
Incremental costs and outcomes	19	For each intervention, report mean values for the main categories of estimated costs and outcomes of interest, as well as mean differences between the comparator groups. If applicable, report incremental cost-effectiveness ratios.	12 (Table 2)
Characterising uncertainty	20a	*Single study-based economic evaluation:* Describe the effects of sampling uncertainty for the estimated incremental cost and incremental effectiveness parameters, together with the impact of methodological assumptions (such as discount rate, study perspective).	13
	20b	*Model-based economic evaluation:* Describe the effects on the results of uncertainty for all input parameters, and uncertainty related to the structure of the model and assumptions.	
Characterising heterogeneity	21	If applicable, report differences in costs, outcomes, or cost-effectiveness that can be explained by variations between subgroups of patients with different baseline characteristics or other observed variability in effects that are not reducible by more information.	13
**Discussion**			
Study findings, limitations, generalisability, and current knowledge	22	Summarise key study findings and describe how they support the conclusions reached. Discuss limitations and the generalisability of the findings and how the findings fit with current knowledge.	15-16
**Other**			
Source of funding	23	Describe how the study was funded and the role of the funder in the identification, design, conduct, and reporting of the analysis. Describe other non-monetary sources of support.	?
Conflicts of interest	24	Describe any potential for conflict of interest of study contributors in accordance with journal policy. In the absence of a journal policy, we recommend authors comply with International Committee of Medical Journal Editors recommendations.	?

A good template page for CHEERS Checklist is as follows: http://www.ispor.org/TaskForces/EconomicPubGuidelines.asp.

Reference:

Husereau D, Drummond M, Petrou S, et al. Consolidated health economic evaluation reporting standards (CHEERS) — Explanation and elaboration: A report of the ISPOR health economic evaluations publication guidelines good reporting practices task force. Value Health 2013;16:231-50.

eTable 2. Search strategy.

Database	Keywords
**Pubmed**	
**(1)**	"nivolumab"[Title/Abstract] OR "pembrolizumab"[Title/Abstract] OR "‘ipilimumab" [Title/Abstract] OR "atezolizumab"[Title/Abstract] OR "camrelizumab"[Title/Abstract] OR "cemiplimab"[Title/Abstract] OR "durvalumab"[Title/Abstract] OR "toripalimab"[Title/Abstract] OR "tislelizumab"[Title/Abstract] OR "PD-1"[Title/Abstract] OR "PD-L1"[Title/Abstract] OR "anti-PD-1"[Title/Abstract] OR "anti-PD-L1"[Title/Abstract] OR "Immune checkpoint inhibitor"[Title/Abstract] OR "ICIs "[Title/Abstract] OR "programmed cell death 1 receptor/antagonists and inhibitors"[MeSH Terms] OR "programmed cell death 1 receptor antagonists and inhibitors"[Title/Abstract] OR "programmed cell death 1 receptor antagonist"[Title/Abstract] OR "programmed cell death 1 receptor inhibitor"[Title/Abstract] OR "Immunotherapy"[Title/Abstract]
**(2)**	"nasopharyngeal cancer"[Title/Abstract] OR "nasopharyngeal carcinoma"[MeSH Terms] OR "NPC"[Title/Abstract]
**(3)**	"clinical trials as topic"[MeSH Terms] OR "clinical trial"[Publication Type] OR "phase II "[Title/Abstract] OR "phase III"[Title/Abstract] OR "phase 2"[Title/Abstract] OR "phase 3"[Title/Abstract] OR "clinical trials"[Title/Abstract]
**(4)**	("2015/01/01"[Date - Publication] : "2021/12/20"[Date - Publication])
**(5)**	(1) AND (2) AND (3) AND (4)
**(6)**	"review"[Article type] OR "meta"[Title] OR "meta-analysis"[Title] OR "protocol"[Title]
**(7)**	(5) NOT (6)
**Embase**	
**(1)**	(nivolumab OR pembrolizumab OR ipilimumab OR atezolizumab OR camrelizumab OR OR cemiplimab OR durvalumab OR toripalimab OR tislelizumab OR tislelizumab OR PD-1 OR PD-L1 OR anti-PD-1 OR anti-PD-1 OR anti-PD-L1 OR anti-PD-L1 OR 'immune checkpoint inhibitors' OR ICIs OR 'programmed cell death 1 receptor/antagonists and inhibitors' OR 'programmed cell death 1 receptor antagonists and inhibitors' OR 'programmed cell death 1 receptor antagonist' OR 'programmed cell death 1 receptor inhibitor' OR Immunotherapy):ti,ab,kw
**(2)**	('nasopharyngeal cancer' OR 'nasopharyngeal carcinoma' OR NPC):ti,ab,kw
**(3)**	trial/exp OR 'clinical trials'/exp OR 'phase 2 clinical trial'/exp OR 'phase 3 clinical trial'/exp OR 'phase II clinical trial'/exp OR 'phase III clinical trial'/exp
**(4)**	[article]/lim OR [article in press]/lim
**(5)**	[humans]/lim
**(6)**	[1-1-2015]/sd NOT [20-12-2021]/sd
**(7)**	(1) AND (2) AND (3) AND (4) AND (5) AND (6) AND (7)
**Cochrane**	

**(1)**	(nivolumab OR pembrolizumab OR ipilimumab OR atezolizumab OR camrelizumab OR cemiplimab OR durvalumab OR toripalimab OR tislelizumab OR tislelizumab OR PD-1 OR PD-L1 OR anti-PD-1 OR anti-PD-1 OR anti-PD-L1 OR 'immune checkpoint inhibitors' OR ICIs OR 'programmed cell death 1 receptor and antagonists and inhibitors' OR 'programmed cell death 1 receptor antagonists and inhibitors' OR 'programmed cell death 1 receptor antagonist' OR 'programmed cell death 1 receptor inhibitor' OR immunotherapy)
**(2)**	('nasopharyngeal cancer' OR 'nasopharyngeal carcinoma' OR NPC)
**(3)**	('clinical trials as topic' OR trial):ti,ab,kw
**(4)**	("conference" OR "review"):pt
**(5)**	Publication date: Between Jan 2012 and Dec 2021
**(6)**	(1) AND (2) AND (3) AND (4) AND (5)

eTable 3. Characteristics of RCTs included in the study.

Study	Phase	Trial name	Total sample size	Drug	Combination	Sample size	Control	Sample size	Median OS (months)	HR for OS (95% CI)	Median PFS (months)	HR for PFS (95% CI)	ORR (%)	Patients with grade 3 or higher AEs, n (%)
Y Yang, 2021	III	CAPTAIN-1st	263	Camrelizumab PD-1	Gemcitabine and Cisplatin	134	Placebo with Gemcitabine and Cisplatin	129	NE vs 22.6	0.67 (0.41 to 1.11)	9.7 vs 6.9	0.54 (0.39 to 0.76)	87.3 vs 80.6	126 (94.0) 118 (91.0)
H Mai, 2021	III	JUPITER-02	289	Toripalimab PD-1	Gemcitabine and Cisplatin	146	Placebo with Gemcitabine and Cisplatin	143	NE vs NE	0.78 (0.37-1.64)	11.7 vs 8.0	0.52 (0.36 to 0.74)	77.4 vs 66.4	130 (89.0) 128 (89.5)

Abbreviation: OS ,Overall Survival; PFS, progression-freesurvival; CI, confidence interval, ORR, Objective Response Rate; AEs, Adverse Events.

eTable 4. Drug dose and cost.

Drug	Dose	Infusion Timing	Unit costs(＄)
Camrelizumab plus gemcitabine and cisplatin	Camrelizumab, 200mg;	six 3-week cycles; followed by maintenance camrelizumab every 3 weeks (a maximum of 2 years of treatment)	2.2208 per 1mg
	Gemcitabine, 1000mg/m^2;		0.1251 per 1mg
	Cisplatin, 80mg/m^2		1.2068 per 1mg
Toripalimab plus gemcitabine and cisplatin	Toripalimab,1500mg;	six 3-week cycles; followed by maintenance toripalimab every 3 weeks (a maximum of 2 years of treatment)	1.3738 per 1 mg
	Gemcitabine, 1000mg/m^2;		0.1251 per 1mg
	Cisplatin, 80mg/m^2		1.2068 per 1mg
gemcitabine plus cisplatin	Gemcitabine, 1000mg/m^2;	six 3-week cycles	0.1251 per 1mg
	Cisplatin, 80mg/m^2		1.2068 per 1mg
Capecitabine	Capecitabine, 1250mg/m^2	3-week cycles, oral administration twice a day (d1-14)	0.0011 per 1 mg

eTable 5. Summary of statistical goodness-of-fit of K-M curve.

	Exponential	Weibull	Gompertz	Log-logistic	Log-normal
**GP OS curve**					
AIC	10.15	11.68	11.83	11.62	11.56
BIC	10.99	13.35	13.50	13.28	13.23
**TGP OS curve**					
AIC	6.66	8.39	8.45	8.38	8.35
BIC	7.55	10.17	10.23	10.16	10.13
**CGP OS curve**					
AIC	7.69	9.51	9.54	9.50	9.47
BIC	8.52	11.17	11.21	11.16	11.14
**GP PFS curve**					
AIC	26.47	36.24	40.09	33.13	33.56
BIC	27.03	37.37	41.22	34.26	34.69
**TGP PFS curve**					
AIC	12.32	14.39	14.50	14.29	14.24
BIC	12.81	15.36	15.47	15.26	15.21
**CGP PFS curve**					
AIC	18.70	22.30	22.52	21.96	21.88
BIC	19.26	23.43	23.65	23.08	23.01

Abbreviation: OS, overall survival; PFS, progression-free survival; AIC, Akaike's information criterion; BIC, Bayesian information criterion.

As for the curves listed in the table, the exponential, log-normal, and log-logistic distribution had the lowest AIC and BIC. However, the exponential, log-normal, and log-logistic models can incorporate non-monotonic hazards but typically have long tails due to a reducing hazard as time increases after a certain point. Actually, the visual fits of the curves (eFigure 3 in the supplementary material) showed that exponential, log-normal, and log-logistic distribution extended tail, which would likely overestimate OS and PFS in the long term based on clinical experts' opinion.

Weibull distributions are flexible and widely used were matched to the number of patients in the three states over time, as its can monotonically increase or decrease the hazard function, it is suitable for estimating the event that occurs in the early follow-up work period. Therefore, the Weibull distributions was likely to be the most reasonable parametric survival model.

eMethods. Network Meta-analysis.

1.1. Study Selection and Assessment of bias risks

This investigation retrieved the Pubmed, Embase, Cochrane, and Web of Science repositories for articles English-written publications from Jan 1, 2015, to Dec 31, 2021, with the search terms “PD-1”, “PD-L1”, “immunotherapy”, “nasopharyngeal carcinoma”, and “clinical trial” (Supplementary Material eFigure 1 and eTable 1). In addition, the investigation also focused on abstracts reported by the American Society of Clinical Oncology (ASCO) and the European Society of Medical Oncology (ESMO). Finally, relevant literature was manually screened to avoid missing articles. Inclusion criteria: (1) Phase III randomized controlled trials (RCTs); (2) compared toripalimab or camrelizumab plus chemotherapy with chemotherapy for adult patients with R/M NPC; and (3) the primary outcomes were OS and progression-free survival (PFS). Studies not matching the inclusion criteria were excluded. YWZ and KL carried out literature retrieval and data extraction independently. Whenever duplicate studies were identified, the article having the most comprehensive and recent investigation data were included. Bias risk assessment of clinical trials was performed using RevMan, Version 5.4.1, according to the guidance provided in the Cochrane manual[1].

1.2. Statistical analysis

We pooled the HR and 95% CI for the OS and PFS of each treatment group in the two RCTs and used R computer program (version 4.1.1, http://www.r-project.org) for comparative analysis. However, as only one RCT involved a pairwise comparison of individuals, and due to the lack of a dataset to assess heterogeneity across the trials, we developed a fixed-effect model[2]. Therefore, the frequency method was employed for the comparison of the comparative effectiveness of various schemes. The HR of OS and PFS, and the corresponding 95% CIs and P-values, were evaluated, and the P-value of each result was used for ranking, where a higher value indicated higher success.

eReferences.

1. Cochrane Training. Cochrane RevMan. Available at: https://training.cochrane.org/onlinelearning/core-software-cochrane-reviews/revman Accessed September 2020. .
2. Rücker G, Schwarzer G. Ranking treatments in frequentist network meta-analysis works without resampling methods. BMC Med Res Methodol 2015; 15: 58.
